# Evolution of Structure and Properties of Nickel-Enriched NiTi Shape Memory Alloy Subjected to Bi-Axial Deformation

**DOI:** 10.3390/ma16020511

**Published:** 2023-01-05

**Authors:** Victor Komarov, Roman Karelin, Irina Khmelevskaya, Vladimir Cherkasov, Vladimir Yusupov, Grzegorz Korpala, Rudolf Kawalla, Ulrich Prahl, Sergey Prokoshkin

**Affiliations:** 1 Baikov Institute of Metallurgy and Materials Science RAS, 119334 Moscow, Russia; 2 National University of Science and Technology MISIS, 119049 Moscow, Russia; 3 Institute of Metal Forming, TU Bergakademie Freiberg, 09599 Freiberg, Germany

**Keywords:** shape memory alloys, NiTi, severe plastic deformation, nanostructured materials

## Abstract

The effect of a promising method of performing a thermomechanical treatment which provides the nanocrystalline structure formation in bulk NiTi shape memory alloy samples and a corresponding improvement to their properties was studied in the present work. The bi-axial severe plastic deformation of Ti-50.7at.%Ni alloy was carried out on the MaxStrain module of the Gleeble system at 350 and 330 °C with accumulated true strains of *e* = 6.6–9.5. The obtained structure and its mechanical and functional properties and martensitic transformations were studied using DSC, X-ray diffractometry, and TEM. A nanocrystalline structure with a grain/subgrain size of below 80 nm was formed in bulk nickel-enriched NiTi alloy after the MaxStrain deformation at 330 °C with *e* = 9.5. The application of MaxStrain leads to the formation of a nanocrystalline structure that is characterized by the appearance of a nano-sized grains and subgrains with equiaxed and elongated shapes and a high free dislocation density. After the MaxStrain deformation at 330 °C with *e* = 9.5 was performed, the completely nanocrystalline structure with the grain/subgrain size of below 80 nm was formed in bulk nickel-enriched NiTi alloy for the first time. The resulting structure provides a total recoverable strain of 12%, which exceeds the highest values that have been reported for bulk nickel-enriched NiTi samples.

## 1. Introduction

One of the most rapidly developing fields of the modern materials science is associated with the smart functional materials with a shape memory effect (SME). NiTi alloys are the most frequently used smart functional materials with the best functional properties compared to all of the shape memory alloys (SMA). Consequently, NiTi SMA are widely applied in the aircraft industry, mechanical engineering, and particularly in medicine [1,2,3]. The most significant medical products manufactured from NiTi SMA are vascular stents, clips of various designs, and devices for orthodontics, etc. [4,5] They are commonly produced from NiTi SMA containing more than 50.5 at.% Ni, which provides shape recovery at body temperature [5,6,7]. The improvement of the functional characteristics of NiTi SMA will improve the reliability and durability of applied devices, and it will also contribute to the design of new ones. Thus, it is a relevant technological and scientific task that requires additional research.

The mechanical and functional properties of NiTi SMA are structure sensitive and depend on the modes of the thermomechanical treatment (TMT) applied for the production of the alloy. Conventional manufacturing technologies of NiTi SMA usually include hot deformation by rolling or forging at recrystallization temperatures and above. Elevated deformation temperatures lead to the formation of the recrystallized structure with a size of about 25–30 microns and a relatively low values of the completely recoverable strain of 4% and lower [8,9]. 

Severe plastic deformation (SPD) is one of the most commonly applied methods of performing a thermomechanical treatment, allowing one to obtain a true nanocrystalline structure (NCS) in NiTi SMA and considerably improve their operational characteristics [10,11,12,13]. However, an NCS can only be obtained in thin NiTi samples after multi-pass cold rolling or high-pressure torsion and subsequent post-deformation annealing (PDA) [10,14]. Therefore, the further development of SPD methods is focused on the search for new deformation modes for manufacturing bulk NiTi samples with NCS [6,7,8].

In our previous studies, the true nanocrystalline structure was formed for the first time in severely deformed bulk NiTi SMA. The application of bi-axial SPD on the MaxStrain (MS) module for the processing of equiatomic Ti-50.0at.% Ni resulted in the development of a mixed nanocrystalline structure, with the average size of the grain/subgrains being 55 nm, and a very high completely recoverable strain of 9% [15,16,17]. The present work continues the previous studies, and it is focused on the investigation of the effect of bi-axial deformation on the structural phase state and properties of bulk nickel-enriched NiTi SMA. In these alloys, deformation in the temperature range of dynamic polygonization is accompanied by dynamic strain aging with the precipitation of Ni_4_Ti_3_ particles, which cannot occur in non-aging equiatomic NiTi SMA, and it has a great impact on the structure’s formation and properties [18,19,20,21]. The precipitated particles restrict the grain growth, however, they can lead to earlier sample destruction compared to that of equiatomic alloy and the accumulation of a lowered value of true strain. Thus, the aim of this work is to continue studying the effect of bi-axial MS deformation on the structure and properties of NiTi SMA, focusing on the nickel-enriched alloy.

## 2. Materials and Methods

Hot-rolled nickel-enriched NiTi (50.7 at.% Ni) SMA plates, with a height of 15 mm, were chosen as the study material. The hot-rolled NiTi rod was subjected to a reference treatment (RT), consisting of annealing at 900 °C for 30 min and subsequent cooling in water.

**Figure 1:**
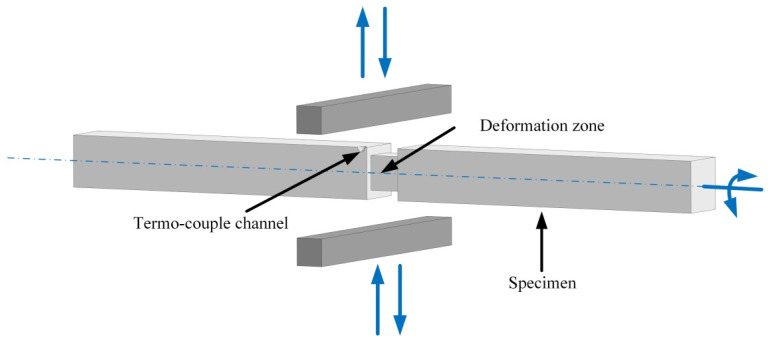
Deformation scheme on the MaxStrain module.

Then, from the reference-treated NiTi plate, samples with a grip zone of 15 × 15 × 150 mm^3^ and a central bulk deformable zone of 10 × 10 × 11 mm^3^ were cut using an electric discharge machine (Figure 1). For the precise control of the temperature change in the deformable zone during the deformation process, a technological channel for the thermocouple was drilled, as is shown in Figure 1. 

Bi-axial MS deformation was performed using the *Gleeble HDS-V40* physical simulation system. During the MS deformation, the central zone of the specimen was deformed by a compression between two strikers with the rotation of the sample by 90° around its longitudinal axis after each strike. The heating of the deformation zone was supplied by the electric current.

In the present study, the following regimes of MS deformation were applied: the deformation cycle was repeated for 10 or 14 times at 350 and 330 °C at an anvil speed of 0.5 mm/s. Accumulated true strains were calculated as the sum of the logarithmic strains for each compression, and these equaled *e* = 6.6 and 9.5.

The samples used for the analyses of the obtained structural phase state and its mechanical and functional properties were cut using the electrical discharge machine. Characteristic temperatures of martensitic transformations (MT) were measured using the *Mettler-Toledo* Differential scanning calorimeter, *DSC-3* with a cooling–heating rate of 10 K/min in the temperature range from −100 to +100 °C. The structure was studied using a *D8 Advance* X-ray diffractometer, a *VEGA 3 TESCAN* Scanning electron microscope (SEM), and a *JEM-2100* Transmission electron microscope (TEM). The samples for the structure analysis were cut from the sample in the direction that was perpendicular to the last compression.

The total recoverable strain $\varepsilon_{rt}$ was determined by employing a thermomechanical method using a bending mode for strain involving cold water (the R-phase state). The size of the sample was 0.5 × 0.5 × 10 mm^3^. The method consists of the following sequence of operations: consecutively increasing the induced strain; determining the value of the residual strain after unloading and the difference between the induced strain and residual strain (elasticity and superelasticity effect); heating it to above the A_s_ temperature for the strain recovery; indicating the values of residual strain and recovered strain (shape memory effect) [9]. The total recoverable strain $\varepsilon_{rt}$ is defined when the residual strain is 0%, and consequently, the shape recovery rate is 100%. The mechanical properties were estimated by the Vickers hardness measurements using the *LECOM 400-A* hardness tester at room temperature under 1N load for 10 s.

## 3. Results and Discussion

### 3.1. MS Deformation

The images of the sample and the scheme of MaxStrain deformation are shown in Figure 1. While MS deformation is biaxial, a certain part of a metal flows out from the deformation zone to the specimen head after each compression due to a large plastic strain component. Reducing the volume of the deformable material makes it difficult to build accurate stress–strain curves, and therefore, we focused on the strain force. During the first compression, the sample was reduced to a height of 7 mm (*e* = 0.36). After the next passes, the reduced height was greater than the previous one was by 0.1–0.2 mm (*e* = 0.6–0.7) in order to ensure uniform strain distribution. The final height of the sample with *e* = 9.5 was 5.5 mm.

The maximum deformation force increased from 160 to 180 kN as the temperature decreased from 350 to 330 °C. During the deformation of the equiatomic NiTi SMA, these values were 15–20 kN lower, which could have been caused both by a change in the phase composition of the alloy and by dispersion hardening associated with the precipitation of the Ni_4_Ti_3_ phase particles.

**Figure 2:**
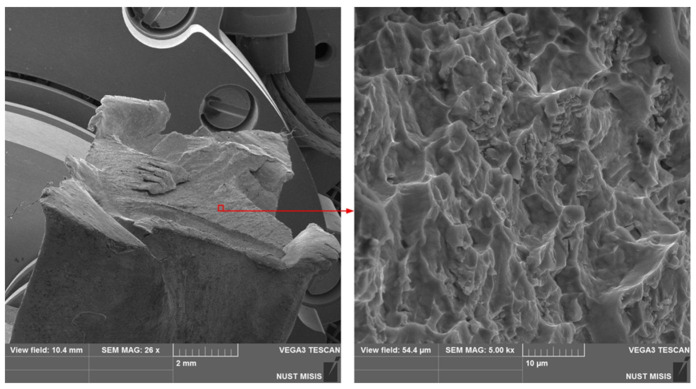
Scanning electron microscopy image of the NiTi SMA sample in the place of destruction after deformation at 330 °C with *e* = 9.5.

At a temperature of 350 °C, the NiTi SMA had good deformability, which allowed it to accumulate the required values of a true strain. The decrease in the deformation temperature to 330 °C was accompanied by an appearance of cracks at the point of transition from the deformed zone to the grip one (Figure 2). Cracks appeared during the last compression at *e* = 9.5, thus, a further increase in the value of accumulated strain at a given temperature was essentially achieved.

The analysis of a fracture reveals a lot of pits of different sizes and depths on the fracture surface (Figure 2). This surface structure is explained by the fact that when the maximum hardened states are reached in local volumes, microvoids appear in areas that are obstacles to continuous deformation. As the stress increases, the microvoids grow and merge, leading to complete destruction, with the formation pits at the fracture, which are interconnected by bridges. Such a pitted microstructure is characteristic of a ductile fracture.

Thus, the critical mode of MS deformation can be defined as deformation at 330 °C with *e* = 9.5. In an equiatomic alloy, the fracture occurred at 250 °C with *e* = 11, which can be explained by the absence of precipitates of the hardening Ni_4_Ti_3_ phase.

### 3.2. XRD Study

**Figure 3:**
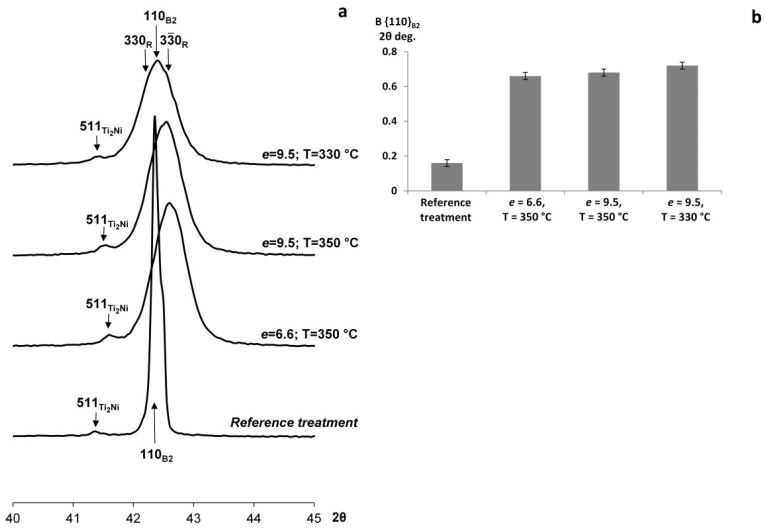
X-ray line{110}_B2_ profiles (**a**) and FWHM (**b**) of NiTi at room temperature after various treatments.

The representative X-ray diffraction patterns of NiTi SMA after the RT and MS deformation, which were obtained at room temperature, are shown in Figure 3a.

The X-ray analysis reveals the B2-austenite line and a certain amount of Ti_2_Ni phase (see {511}_Ti_2_Ni_ line) at room temperature after the RT. Deformation leads to an increase in the characteristic temperatures of martensitic transformation and the corresponding appearance of the intermediate R-phase at room temperature. This increase is proven by the results of DSC.

The measurements of the X-ray line width of B2-austenite B_hkl_ (full width at half maximum (FWHM)) allow us to estimate changes in the crystal lattice’s defectiveness. MS deformation is accompanied by a dramatic increase in the B_110_ line FWHM, which indicates a significant increase in the degree of the lattice’s defectiveness and the appearance of the R-phase (Figure 3b). The R-phase may contribute to the line broadening, but considering the observed changes, this contribution can be omitted.

### 3.3. TEM Study

In the studied alloy, after the RT, the average size of the austenite grains is about 25–30 microns. To study the structure and phase composition of the NiTi alloy, the TEM images were obtained after MS deformation with accumulated strains of *e* = 6.6 and 9.5 at 350 and 330 °C, respectively. In the temperature range of 300–500 °C, the dynamic polygonization processes must develop in the nickel-enriched NiTi SMA, as was shown in [18].

**Figure 4:**
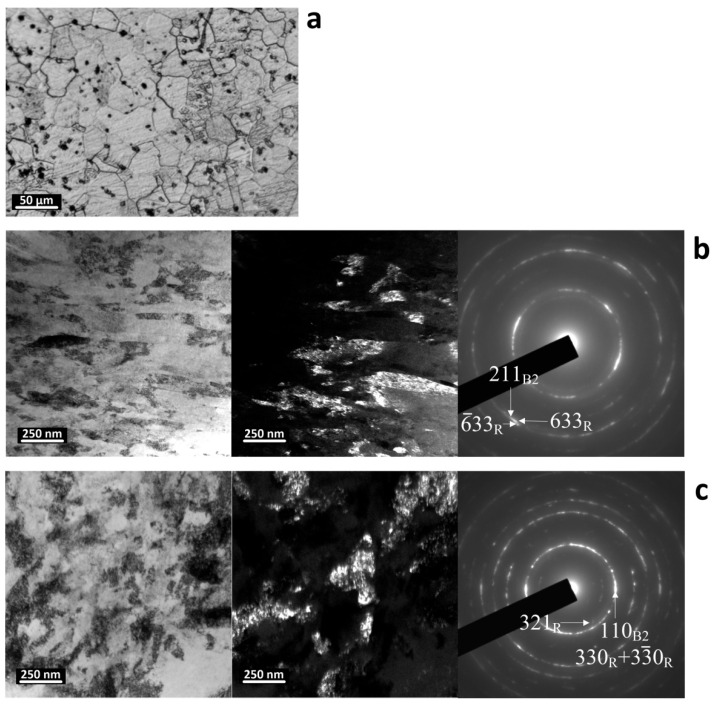
Structure of NiTi alloy after reference treatment, light optical microscopy (**a**), after MS deformation at 350 °C, *e* = 6.6 (**b**), and 330 °C, *e* = 9.5 (**c**); both of them involved transmission electron microscopy. Left, bright-field images; center, dark-field images; right, selected area electron diffraction patterns.

The bright and dark field TEM images reveal that MS deformation at 350 °C leads to the formation of a nanocrystalline structure that is characterized by the appearance of a nano-sized grains and subgrains (50–100 nm in transverse direction) with equiaxed and elongated shapes of about 10^11^ cm^−2^ and a high free dislocation density (Figure 4). The cellular substructure and submicron-sized areas with a uniform distribution of dislocations can be observed as well. It can be consequently concluded that the dynamic recovery and polygonization processes in nickel-enriched NiTi SMA in the same thermomechanical conditions are less developed compared to those of an equiatomic NiTi alloy. This can be explained by the retarding effect of the finely dispersed Ni_4_Ti_3_ phase particles that were precipitated during dynamic strain aging [19,20].

After the analysis of the SAED pattern, only the reflexes of B2-austenite and weak reflexes of the R-phase were determined. The observed parallel plates are crystals of the R-phase, which are formed upon cooling after MS deformation. The TEM study does not reliably reveal the reflexes or images of the Ni_4_Ti_3_ phase due to there being a very high degree of lattice defectiveness and extremely small Ni_4_Ti_3_ particle sizes.

MS deformation at 330 °C, *e* = 9.5, also leads to the formation of the nanograined/nanosubgrained structure in combination with a highly dislocated substructure (Figure 4b). The dislocation density in this case is visually higher than it is after the MS deformation at 350 °C; the grains/subgrains are more equiaxed and smaller. Thus, under MS deformation at 330 and 350 °C, the processes of dislocations redistribution are limited due to the retarding effect of the Ni_4_Ti_3_ phase particles, and the nanograined/nanosubgrained structure is less developed than it is in the non-aging NiTi alloy [16,17].

### 3.4. DSC Study

**Figure 5:**
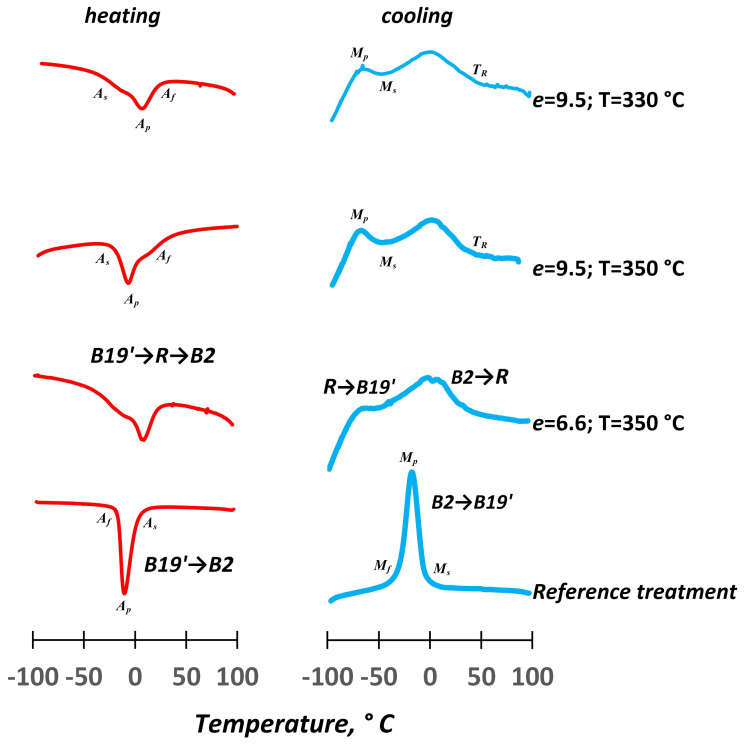
DSC curves of NiTi at room temperature after various treatments.

The results of the DSC show that after the RT, one-stage B2 ↔ B19′ forward and reverse MTs proceed, which are indicating by a single calorimetric peak on the calorimetric curves (Figure 1). The obtained characteristic temperatures of the MT are presented in Table 1: *Ms*, *M_f_* and *A_s_*, *A_f_* are start and finish temperatures of the forward and reverse MTs; *T_R_*—starting temperature of B2→R MT; *M_p_*; *A_p_*—peak temperatures of forward and reverse MTs. The increase in the strain leads to the appearance of a two-stage B2→R→B19′ transformation, which is indicated by the broadening and multiplication of the peaks in both of the MTs (Figure 5; Table 1).

The strain hardening in combination with the strain aging has the highest influence on the MT sequence and temperatures during the MS deformation. The formation of B19′-martensite is inhibited by the strain hardening which also facilitates the formation of the R-phase from B2-austenite. The dynamic precipitation of Ni_4_Ti_3_ phase particles indicates, indirectly, significant shifts in the temperature range of the forward martensitic transformation which are below minus 100 °C and cannot be explained only by the deformation hardening. The formation of the Ni_4_Ti_3_ phase particles, because of the strain aging, also inhibits the R-phase formation. The increase in the strain rate from 6.6 to 9.5 at 350 °C does not significantly change the calorimetric peak positions.

### 3.5. Functional Properties and Hardness

The strain hardening of the alloys induced by MS deformation increases the Vickers hardness and the total completely recoverable strain $\varepsilon_{rt}$, which is a main functional property of SMA (Table 2). The higher the resistance is to plastic deformation, the higher the strain is that can be induced without involving a dislocation slip and then recovered by shape memory mechanisms, such as thermoelastic MT and martensite reorientation [22].

The MS deformation is accompanied by significant increase in hardness compared to that which occurs during the RT (Table 2). This trend continues with an increase in the accumulated strain and a decrease in the deformation temperature. We note that the hardness of the previously studied equiatomic alloy is lower compared to that of the Ti-50.7at.%Ni alloy by 15–30 HV under the same MS deformation conditions [18]. This result may be caused by different deformation mechanisms involved in the indentation process. In the nickel-enriched NiTi SMA, indentation is performed at room temperature, i.e., which is far above the *A_S_* temperature, and recovery upon unloading includes the elastic and superelastic strain components, whereas the equiatomic alloy indented below *M_f_* undergoes only elastic recovery upon unloading [22].

The calorimetric study of the Ti-50.7at.%Ni alloy shows that MS deformation leads to the change in the MT sequence, which proceeds through the intermediate R-phase, and the significant extension of the R-phase(Table 1). Therefore, during $\varepsilon_{rt}$ measurement, the strain induction starts in the R-phase state. Such a strain-inducing condition allows it to reaching maximum values of the recoverable strain compared to that of the strain-inducing condition in the B19′-martensite state because of the difference between the critical stress for B19′-martensite formation in the R-phase (transformation yield stress) and the reorientation stress of thermal B19′-martensite.

The $\varepsilon_{rt}$ value consists of three components: elastic strain and superelastic strain, which recover upon unloading, and the recovery strain of the shape memory effect, which recovers while heating it above *A_f_*. The results of the $\varepsilon_{rt}$ measurement for Ti-50.7at.%Ni alloy are shown in Table 2. After all of the studied regimes of the MS deformation were conducted, the $\varepsilon_{rt}$ was 11–12%, which is comparable with the highest value obtained in previous studies [13,17,22], and it is much higher compared to that of the RT.

The total recoverable strain’s tendency to increase with an increase in the accumulated strains and decrease in the MS deformation temperatures correlates with the increase in the hardness, which indicates an increase in the MS deformation hardening (Table 2). This tendency has also been observed earlier for the equiatomic NiTi alloy [13,16,17,18]. The increase in hardness is accompanied by the increase in the dislocation yield stress. Therefore, the difference between the dislocation and transformation yield stresses increases as the accumulated strain increases and the deformation temperature decreases. This leads to the later involvement of the irrecoverable dislocation slip in the deformation process, and consequentially, an improvement of the shape recovery characteristics [17,22].

## 4. Conclusions

The severe plastic deformation of the nickel-enriched Ti-50.7at.%Ni shape memory alloy was carried out for the first time using the MaxStrain (MS) mode at temperatures of 350 and 330 °C with accumulated true strains of *e* = 6.6 and 9.5, respectively. The critical regime of MS deformation accompanied by the destruction of a sample was defined as T = 330 °C, with *e* = 9.5. As a result of MS deformation, a nanocrystalline structure with a high dislocation density and an average grain/subgrain size that was below 100 nm was formed, providing a significant increase in the total completely recoverable strain of the nickel-enriched NiTi SMA of up to 12% compared to 4% which occurred after the reference treatment. Traces of the Ni_4_Ti_3_ phase particles were not observed directly by the TEM and X-ray studies because of their extremely fine size, which were presumably less than 5 nm. However, a dynamic deformation-induced precipitation of Ni_4_Ti_3_ phase particles was indicated indirectly by the DSC results. After the MS deformation at 330 and 350 °C of nickel-enriched NiTi alloys, the processes of dynamic recovery and polygonization, including dislocation redistribution and subgrains formation, were limited due to the retarding effect of the Ni_4_Ti_3_ phase particles, and the nanograined/nanosubgrained structure was less developed compared to that of the near-equiatomic NiTi alloys. 

## Figures and Tables

**Table 1:** Characteristic temperatures of martensitic transformation of NiTi SMA.

Treatment	*M_f_*, °C	*M_p_*,°C	*M_s_*, °C	*T_R_*,°C	*A_s_*, °C	*A_p_*,°C	*A_f_*, °C
Reference	−32	−17	−4	-	−20	−13	1
T = 350 °C, *e* = 6.6	<−100	−70	−40	45	−26	−10	42
T = 350 °C, *e* = 9.5	<−100	−68	−32	45	−22	−8	35
T = 330 °C, *e* = 9.5	<−100	−70	−35	49	−23	−8	41

**Table 2:** Hardness and total maximum completely recoverable strain of NiTi SMA.

Treatment	$\bar{HV_{1}}$	$\varepsilon_{rt}$
Reference	242 ± 4	4.0 ± 0.3
T = 350 °C, *e* = 6.6	325 ± 7	10.9 ± 0.3
T = 350 °C, *e* = 9.5	341 ± 7	11.7 ± 0.3
T = 330 °C, *e* = 9.5	362 ± 6	12.0 ± 0.3

## Data Availability

Not applicable.
